# Is spurious penicillin allergy a major public health concern only in high-income countries?

**DOI:** 10.1136/bmjgh-2021-005437

**Published:** 2021-05-20

**Authors:** Mamidipudi Thirumala Krishna, Pudupakkam K Vedanthan, Rajesh Vedanthan, Reham Mohamed El Shabrawy, Ramesh Madhan, Hoa L Nguyen, Thushara Kudagammana, Iestyn Williams, Biraj Karmacharya, Seetharaman Hariharan, Kandamaran Krishnamurthy, Stevent Sumantri, Rachel Elliott, Padukudru Anand Mahesh, John F. Marriott

**Affiliations:** 1 Department of Allergy and Immunology, University Hospitals Birmingham NHS Foundation Trust, Birmingham, West Midlands, UK; 2 Institute of Immunology and Immunotherapy, University of Birmingham, Edgbaston Campus, Birmingham, UK; 3 Department of Medicine, University of Colorado at Anschutz campus, Aurora, Oregon, USA; 4 Department of Population Health, NYU Grossman School of Medicine, New York, New York, USA; 5 Faculty of Medicine, Zagazig University, Zagazig, Egypt; 6 Department of Phamacy Practice, JSS Academy of Higher Education and Research, Mysore, Karnataka, India; 7 Division of Epidemiology, Department of Population and Quantitative Health Sciences, University of Massachusetts Medical School, Worcester, Massachusetts, USA; 8 Department of Paediatrics, Faculty of Medicine, University of Peradeniya, Peradeniya, Central, Sri Lanka; 9 Health Services Management Centre, University of Birmingham, Birmingham, West Midlands, UK; 10 Departments of Public Health and Community Programs, Dhulikhel Hospital Kathmandu University Hospital, Kathmandu, Nepal; 11 Department of Clinical Surgical Sciences, The University of the West Indies St Augustine Campus, St Augustine, Tunapuna-Piarco, Trinidad and Tobago; 12 Department of Pediatrics, The Queen Elizabeth Hospital, Bridgetown, Saint Michael, Barbados; 13 Department of Internal Medicine, Universitas Pelita Harapan, Tangerang, Banten, Indonesia; 14 Division of Population Health, Health Services Research and Primary care, The University of Manchester, Manchester, UK; 15 Department of Respiratory Medicine, JSS Medical College, JSSAHER, Mysore, Karnataka, India; 16 The School of Pharmacy, University of Birmingham, Birmingham, UK

**Keywords:** health education and promotion, health systems, medical microbiology, public health

**Summary box**

- Inaccurate penicillin allergy labels (PALs) are a major public health problem in high-income countries and has been linked to antimicrobial resistance (AMR) and huge healthcare costs.
- Data regarding epidemiology of PALs and its potential association with AMR is sparse in low-income countries (LICs), low-middle-income countries (LMICs) and upper-middle-income countries (UMICs).
- There are no established drug allergy labelling and delabelling pathways in the majority of the LICs, LMICs and UMICs and addressing these inequities is critical for safe clinical practice and in the global campaign against AMR.
- A standardised validated computerised decision support tool might help address these gaps, but understanding local factors including clinical governance, cultural, social, religious and human behaviour will be key to uptake and success of such an intervention.

## Introduction

Penicillins are the most common antibiotic class prescribed globally in primary, secondary and tertiary care for common and serious bacterial infections, including sepsis. They are cost-effective medications, available in parenteral and oral formulations and have an excellent safety profile. The downside to the use of penicillins is that they are most commonly implicated in adverse drug reactions including allergy or hypersensitivity, the latter owing to a greater chance of becoming sensitised following frequent exposure.

## The burden and adverse impact of penicillin allergy labels in high-income countries

Penicillin allergy labels (PALs) occur in 6% and 10% of the general population in England and USA, respectively,^1 2^ and in 15%–20% of inpatients in USA^3^ and England (local audit data). Importantly, 90%–95% of PALs are spurious (or inaccurate) and present a significant impediment to prompt and effective antimicrobial stewardship.^1^ This is related to multiple factors including gaps in knowledge and skills among prescribers, and inaccurate documentation and interpretation of side effects leading to mislabelling as penicillin allergy.^4 5^ PALs lead to increased prescription of alternative second line antibiotics such as quinolones, glycopeptides and carbapenems. Apart from increased antibiotic costs, this enhances the risk of antimicrobial resistance (AMR) including methicillin resistant *Staphylococcus aureus*, iatrogenic infections such as *Clostridioides difficile* and vancomycin resistant *Enterococcus*, surgical site infections, delayed treatment of sepsis and contributes to longer hospital stay.^6–9^ To put AMR into a global perspective, it results in an estimated 700 000 deaths/year and the burden is higher in low-income countries (LICs; as per World Bank classification; gross national income (GNI) per capita on 1 July 2020, <US$1036) and low-middle-income countries (LMICs; GNI, US$1036–US$4045).^10 11^ It has been suggested that if there are no mitigation strategies in place to address AMR by 2050, an estimated loss of 10 million lives alongside a cost of US$100 trillion has been predicted.^10^ The United Nations, in its 2016 resolution declared AMR as a ‘high priority’ area.

The excess costs incurred owing to spurious PALs have been estimated to be in the order of several million US dollars each year.^6^

The adverse impact of inaccurate PALs is compounded by the lack of a reliable ‘point-of-care’ test for the diagnosis of penicillin allergy. The current approach for penicillin allergy investigations includes a comprehensive clinical history, allergy skin tests, and if negative, a supervised oral penicillin challenge (gold standard to confirm clinical tolerance). These are onerous, time consuming and require specialist expertise in drug allergy, which is not available in the majority of countries.^12^ Hence, penicillin allergy testing is not available in routine clinical practice, including in most high-income countries (HICs; GNI, >US$12 535), and this often leads to default prescription of second line antibiotics with increased potential for AMR.

## Lessons learnt from penicillin allergy research in HICs

There has been great interest and strides made in a new service model to de-label (removing incorrect labels) spurious penicillin allergy. This involves two methods employing a multiprofessional approach involving experts in drug allergy, infectious diseases, internal medicine, microbiology and pharmacy.^3^ The first is ‘direct penicillin allergy delabelling’ (ie, removing incorrect labels from patient records based on clinical history alone; in those where allergy can be confidently excluded without further intervention). The second is ‘direct oral penicillin challenge’ (DPC), which involves administration of penicillins orally (either as a single dose or graded administration) under close clinical supervision to patients who are least likely to be allergic (eg, those reporting non-specific symptoms such as mild rash, headache or diarrhoea) based on clinical risk stratification and without undertaking allergy testing.^6 13^ In cases where penicillin allergy is likely, a specialist referral is recommended for allergy skin tests.^6^

Facilities for conducting a DPC are usually available in most clinical settings in secondary care, but engagement of non-specialists in conducting this procedure might be hindered by lack of adequate training in drug allergy history taking and concerns related to the rare complication of provoking anaphylaxis. A strategic approach of stratification into ‘low risk’ and ‘high risk’ (history suggestive of anaphylaxis or other serious reactions) has proven successful in research conducted in USA and Australia.^6 14^ It has been reported that up to 60% of patients can be stratified as ‘low risk’ and are suitable for a DPC, and 80% of this cohort would be agreeable to this procedure leading to removal of up to 50% of inaccurate PALs. The Partners Healthcare system, a group of five hospitals in Boston, USA developed a computerised decision tool that is made available to a non-specialist prescriber, with an aim to enhance antimicrobial stewardship and quality of care.^6^ This digital tool processes historical clinical information to enable stratification as ‘low risk’ or ‘high risk’, in order to safely delabel patients with inaccurate PALs without undertaking allergy testing. Their approach resulted in a twofold increase in chances of receiving a beta-lactam antibiotic for inpatients with a PAL, with a projected overall cost saving of US$8.9–US$13.7 million each year.^6^ Importantly, they did not report any serious adverse events employing this approach. Similar e-tools for penicillin allergy delabelling have also been developed by other research groups.^3 14^ Qualitative research conducted in the UK suggested that patients and healthcare professionals would accept such an approach in the National Health Service, if undertaken in a safe clinical environment while employing validated tools.^15^ Recent studies have also shown that penicillin allergy delabelling could be facilitated by nurses and clinical pharmacists in secondary care with support from clinicians, thus allowing greater flexibility in service delivery.^1^

In order to understand how the learning from such developments might be applied to healthcare in LICs, LMICs and upper-middle-income countries (UMICs; GNI, US$4046–US$12 535) settings, it is important to address the following questions:

- What is the burden of PALs in the range of LICs, LMICs and UMICs?
- If so, what would be the clinical and economic impact of reducing this burden?
- How important is PAL as a risk factor to AMR in LICs, LMICs and UMICs?
- What are the views and perspectives of patients and healthcare workers in these countries with regards to PALs?
- What is a feasible, acceptable, effective and affordable solution for PALs in these countries?
- What are the common and unique factors that should be considered when developing interventions to reduce inaccurate PALs and aid antimicrobial stewardship?

## Relevance to LICs, LMICs and UMICs

At present, there are no reliable data regarding the epidemiology of PALs, particularly in LICs and LMICs and it is difficult to estimate the burden of the problem owing to multiple variables contributing to PALs including health service framework and financing, prescribing patterns, availability of antibiotics to patients without a valid prescription, antimicrobial stewardship policy, data capturing systems, ethnicity, cultural, religious and social factors, education and training and as yet unknown factors.

While recent studies from the USA and UK have shown that PALs significantly enhance the risk of AMR,^1 7^ there are no data available at present regarding such an association in LICs and LMICs. The WHO Global Action Plan 2019 (WHO | Global action plan on AMR) lists five objectives including optimisation of medicines use in human and animal health (objective 4). However, tackling inaccurate PALs is not specifically addressed under this objective.

It is recognised that health systems and services are relatively weaker in LICs and LMICs. There is significant heterogeneity with respect to health service funding (eg, free care vs subsidiaries) by government, socioeconomic strata of patients, affordability of medicines and a wealthy section of the population having access to corporate or private hospitals as well as other local factors as described above. Allergy and immunology is a relatively small medical discipline in HICs and has not yet gained specialty recognition in the majority of LICs and LMICs, which comprise a vast amount of the global population. Therefore, prescribers in these countries are not formally trained in basic concepts of drug allergy and there is a lack of clinical leadership in the field of allergy leading to huge gaps in healthcare.

There is an urgent need to generate reliable data regarding the burden of PALs (and other antibiotic allergies) in LICs, LMICs and UMICs and to understand any adverse impact on clinical outcomes including AMR and related health system costs.

## Way forward

Given the heterogeneity in healthcare frameworks in LICs, LMICs and UMICs, a ‘one size fits all’ approach is unlikely to be successful. A multipronged, multimodality and multiprofessional approach involving patient representatives and policy-makers including a combination of epidemiological, qualitative (focus group discussions and semistructured interviews among stakeholders including health leaders, health providers, community members and patients), health economic and implementation science research is urgently needed to develop ‘fit for purpose’ strategies in LICs, LMICs and UMICs. This should be supported by education and training in basic aspects of drug allergy for clinicians and other healthcare professionals involved in prescribing and administration of medicines. This strategy should also be supplemented by education materials for patients and their carer’s.

**Figure 1:**
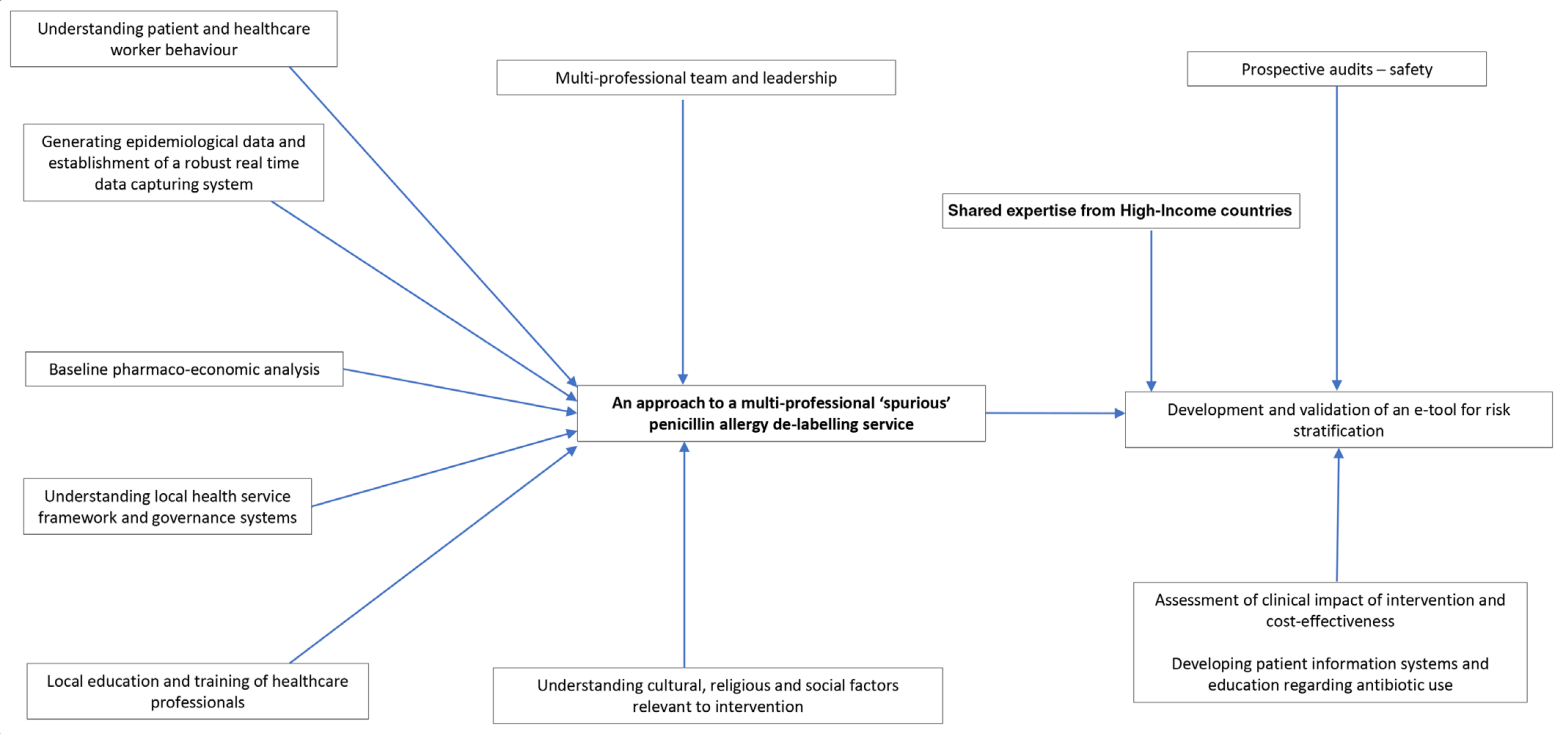
A schematic diagram illustrating a systematic multipronged approach to address penicillin allergy delabelling in low-income, low-middle-income and upper middle-income countries.

It is likely that a computerised decision tool accessed via portable e-devices to assist healthcare professionals to rapidly capture and process historical clinical information to risk stratify and de-label patients will also be successful in these countries. Accommodating and adapting to critical factors relevant to local clinical practice as well as governance frameworks will be key to maximising uptake of such an intervention. Specialists in HICs have an important role to help set up virtual learning environment platforms for healthcare professionals in LICs, LMICs and UMICs and this would need pump priming resources and a collaborative approach, with a sound understanding of facilitators and barriers. Figure 1 summarises a strategic approach to development of a new service model for inaccurate penicillin allergy delabelling in LICs, LMICs and UMICs.

## Conclusion

Current evidence suggests that PALs are undoubtedly a major public health problem in HICs. The data are, however, sparse in LICs, LMICs and UMICs and there is an urgent need for further studies to address this very important gap in clinical medicine. Understanding the epidemiology of PALs alongside its potential association with AMR and high health service costs in these countries will pave the way for development of robust and concerted global strategies for high quality antimicrobial stewardship.
